# The Effect of an Optimized Diet as an Adjunct to Non-Surgical Periodontal Therapy in Subjects with Periodontitis: A Prospective Study

**DOI:** 10.3390/healthcare10030583

**Published:** 2022-03-21

**Authors:** Paolo De Angelis, Giulio Gasparini, Paolo Francesco Manicone, Pier Carmine Passarelli, Domenico Azzolino, Edoardo Rella, Giuseppe De Rosa, Piero Papi, Giorgio Pompa, Silvio De Angelis, Roberta Grassi, Antonio D’Addona

**Affiliations:** 1 Division of Oral Surgery and Implantology, Department of Head and Neck, Institute of Clinical Dentistry, Oral Surgery and Implantology Unit, Fondazione Policlinico Universitario A. Gemelli IRCCS—Università Cattolica del Sacro Cuore, 00168 Rome, Italy; dr.paolodeangelis@gmail.com (P.D.A.); paolofrancesco.manicone@unicatt.it (P.F.M.); piercarmine.passarelli@unicatt.it (P.C.P.); giuseppederosa1410@gmail.com (G.D.R.); antonio.daddona@unicatt.it (A.D.); 2 Independent Researcher, 63100 Ascoli Piceno, Italy; silvio.deangelis@gmail.com; 3 Division of Oral and Maxillo-Facial Surgery, Department of Head and Neck and Sensory Organs, Fondazione Policlinico Universitario A. Gemelli IRCCS—Università Cattolica del Sacro Cuore, 00168 Rome, Italy; giulio.gasparini@unicatt.it; 4 Department of Clinical and Community Sciences, University of Milan, 20122 Milan, Italy; domenico.azzolino@unimi.it; 5 Department of Oral and Maxillo-Facial Sciences, Sapienza University of Rome, 00168 Rome, Italy; piero.papi@uniroma1.it (P.P.); giorgio.pompa@uniroma1.it (G.P.); 6 Department of Oral Surgery, Tor Vergata University, 00133 Rome, Italy; robertagrassi@gmail.com

**Keywords:** gingiva, inflammatory response, oral cavity, periodontitis, diet, fatty acids

## Abstract

Diet and nutrition are generally categorized as modifiable lifestyle risk factors for the development of periodontal disease because diet may influence a person’s inflammatory status. This study aimed to evaluate the efficacy of the application of a diet plan focused on reducing inflammation and oxidative stress in treating periodontitis. Subjects suffering from periodontitis were divided into two groups. Both groups underwent non-surgical periodontal therapy, and in the optimized diet (OD) group, this treatment was associated with a diet plan. The sample consisted of 60 subjects; 32 (53%) were treated in the non-optimized diet group (ND group) and 28 (47%) in the OD group. In both groups, the periodontal treatment significantly improved the recorded periodontal outcomes between T0 and T1 (FMPS, FMBS, CAL, PPD). Inter-group differences were not statistically significant (*p* < 0.05). The linear regression models showed that the optimized diet was associated with a higher reduction in PPD and FMBS after the treatment, while patients who had higher LDL levels (over 100 mg/mL) had a less favorable improvement of PPD. The application of an improved diet plan can increase the reduction in PPD and FMBS after non-surgical periodontal therapy when compared with periodontal treatment alone.

## 1. Introduction

Periodontitis is a common chronic inflammatory disease affecting 10–15% of the developed world’s population. Chronic periodontitis is a multifactorial disorder; however, microbial dental plaque biofilms are considered the main etiological factor [1].

The necessary factor for the development of this disease is the overgrowth of biofilm, especially in the subgingival area [2], but the presence of other systemic and local factors can have a fundamental role in the pathogenesis of this disease [1].

The role of risk factors should be carefully considered because they can change the susceptibility or resistance of individuals to the disease [2,3]. Systemic risk factors for periodontal disease include behaviors, such as smoking, medical conditions (e.g., poorly controlled diabetes, obesity, stress, osteopenia), and inadequate dietary consumption of calcium and vitamin D [2]. Recently, obesity and low levels of physical activity were recognized as risk factors for periodontitis [4].

Macronutrients and micronutrients modulating pro-inflammatory and anti-inflammatory cascades can affect a person’s baseline inflammatory status. Nutrients do not only provide cofactors for metabolism but can also modify proteins and gene expression [5].

Diets based on complex carbohydrates are generally healthy, whereas those rich in refined carbohydrates can be more associated with chronic inflammation. Exaggerated postprandial surges in glucose (and triglycerides) result from high-calorie diets, especially from diets containing refined and processed foods rich in glucose and lipids that can be absorbed rapidly into the bloodstream.

Increasing evidence suggests that micronutrient deficiencies may have a negative impact on periodontal status [6,7]. It is well known that several micronutrients (e.g., vitamin C, β-carotene, vitamin E) found in some fruits, vegetables, grains and seeds share antioxidant properties counteracting both oxidative stress and inflammation [8,9]. Intake of vitamins C and D has been suggested to have a positive impact on periodontal health [10]. Interestingly, Vitamin C deficiency (i.e., scurvy) has been linked historically to periodontitis because it is accompanied by massive periodontal bone loss [9,10]. Many studies in the last decades have found that diets focused on plant-based low-carbohydrate foods and paleo diets can reduce the inflammatory status [11,12]. If confirmed, intervention studies involving antioxidant approaches would be indicated to determine the potential for reducing the risk of periodontitis [13]. These applications should be carefully evaluated as obtaining a better outcome in treating periodontitis could possibly improve many systemic diseases that have been linked to periodontal disease, such as diabetes and cardiovascular disease [14], as it has similarly been observed that periodontitis might be exacerbated by many systemic disorders other than obesity, such as rheumatoid arthritis and osteoporosis [15,16].

Therefore, the objective of this study is to evaluate the efficacy of the application of a diet plan focused on reducing inflammation and oxidative stress in treating periodontitis by monitoring patients for 6 months after non-surgical periodontal therapy.

## 2. Materials and Methods

The study was performed as a prospective clinical trial, with a 6-month follow-up. Subjects were recruited from a private dental clinic in Ascoli Piceno, Italy, between 1 March 2020 and 1 October 2020.

All procedures were performed according to the Declaration of Helsinki guidelines on experimentation involving human subjects. Each participant enrolled in the study received explanations about the study design and objectives and provided written informed consent. Ethical approval was obtained from the institutional review board of the Department of Oral and Maxillo-Facial Sciences at Sapienza University, Rome.

Subjects who were at least 18 years old and suffered from periodontitis according to the criteria of the 2017 classification of the American Academy of Periodontology and the European Federation of Periodontology were included in this study.

Patients with uncontrolled systemic diseases that could have impeded the application of the diet plan (e.g., cancer) or previous head or neck radiation therapy were excluded. In addition, pregnant or lactating females and patients treated with bone-modifying medications or drugs influencing gingival inflammation or bleeding (e.g., anticoagulants, cortisone) or anti-inflammatory drugs were excluded. Finally, any patient who was unable to perform adequate oral hygiene, was a current smoker, had received any periodontal treatment in the prior 6 months, or had been taking systemic antibiotics in the past 3 months was also excluded.

All recruited subjects were submitted to a pre-operative complete periodontal examination, including a full medical and dental history, an intraoral examination, a full-mouth periodontal probing, and a full-mouth intraoral radiographic examination (if a recent one taken in the last 6 months was not available). Sociodemographic factors (age, sex, race) also were recorded. Anthropometric data, including weight (in kilos), height (in meters), and body mass index (BMI), calculated as patient’s weight in kg divided by patient height squared, were measured. Obesity was defined (Y/N) as a BMI ≥ 30 kg/m^2^. In addition, blood pressure was measured with a mercury sphygmomanometer after the patient had been lying supine for 5 min.

Simultaneously, a lipid profile was required and triglycerides (TGR), total cholesterol, high-density lipoprotein (HDL) cholesterol and low-density lipoprotein (LDL) cholesterol were recorded at baseline.

Participants were assigned to one of two groups: Optimized diet (OD) group and non-optimized diet group (ND). Both groups underwent non-surgical periodontal therapy and in the OD group, this treatment was associated with an optimized diet plan.

The decision as to which of the two protocols to perform was made after a discussion with the patient. Patients who refused to undergo an optimized diet were treated with non-surgical periodontal therapy only.

Periodontal therapy was initiated within 1 month from the baseline screening examination.

A standard cycle of non-surgical periodontal therapy was performed in all patients by a single experienced therapist and carried out into two sessions of one hour each. Supra- and sub-gingival mechanical instrumentation was performed with a piezoelectric instrument with fine tips (EMS, Nyon, Switzerland) and hand curettes. Local anesthesia was used as necessary. All patients received detailed dental hygiene instructions on correct toothbrushing, together with interdental cleaning procedures, and was told to conduct this hygiene routine twice a day. According to their specific periodontal status, one or two different interdental brushes sizes were directly provided to each patient.

Subjects in the OD group were provided with a 4-week diet program characterized by the following elements:

- Low carbohydrate content (<130 g/day or <26% of total energy intake). This includes restriction of the amount of fructose, disaccharides, sweetened beverages and meals, flour-containing foods, and pasta, rice, bread and potatoes. There were no restrictions regarding fruits and vegetables (polysaccharides) as long as the total amount of carbohydrates was considered.
- Daily intake of whole-grain products and fiber (whole-grain breakfast cereals, whole-wheat pasta, whole-grain bread, vegetables and fruits)
- Daily intake of omega-3 fatty acids (fish oil capsules, a portion of fatty fish, two spoons of flaxseed oil); a restriction in the amount of trans-fatty acids as far as possible (fried meals, crisps, donuts, croissants); daily intake of monounsaturated fatty acid (olive oil); and a reduction in omega-6 fatty acids as far as possible (such as safflower oil, grape seed oil, sunflower oil, margarine, sesame oil, corn oil)
- Daily intake of a source of vitamin C (kiwis, oranges, bell peppers, etc.)
- Daily intake of a source of vitamin D (15 min unprotected sun exposure, fatty fish, cod liver oil, supplementation with 500 international units (12.5 μg))
- Daily intake of antioxidants (such as a handful of berries, olive oil, or a cup of green tea).

Total energy needs were estimated based on resting energy expenditure (REE) multiplied by physical activity level (PAL) [11]. Energy intake was individually adjusted based on nutritional status, clinical condition (i.e., disease status, masticatory function) and tolerance. Detailed dietary recommendations containing an additional list of restricted and recommended foods were delivered both verbally and in written form. If more information was needed, participants could contact the research team at any time during the study. Appropriate adjustments were made as needed to ensure high compliance.

After 4 weeks of dietary intervention, participants in the test group received detailed dietary advice on how to continue their diet. This included:

- Simple sugars restriction (e.g., confectionery, sweetened beverages, disaccharides, etc.)
- Daily consumption of whole-grain products (e.g., whole-grain cereals, wholewheat pasta)
- Daily consumption of fruit and vegetables
- Consumption of omega-3 fatty acids sources (e.g., fatty fish, flaxseeds) with concomitant reduction of saturated, trans- and omega-6 fatty acids (e.g.,, butter, margarine).

Dietary intake was assessed using a three-day food diary immediately after the intervention and at the end of the intervention to assess both compliance and dietary intake.

Participants were asked to keep consecutive 3-day food records, including one weekend day and two weekdays, to capture day-to-day variability. Participants were given oral and written instructions about compiling a food diary and how to weigh foods and drinks. If they found difficulties in weighing food, participants were asked to use household equipment (e.g., glasses, spoons, cups) facilitated by photographs showing portion sizes and their corresponding weights. Use of vitamin and mineral supplements was also recorded. Food records were reviewed in detail with each participant to locate unclear descriptions, errors, omissions, or doubtful entries and participants were asked to clarify them. Quantification and analysis of energy and nutrient intake were performed using computer software (MètaDieta^®^, ME.TE.DA. S.R.L., San Benedetto del Tronto, Italy).

The primary outcome of the present study was to observe the effects of an optimized diet plan on periodontitis clinical parameters when compared to conventional periodontitis treatment; the secondary outcome was to relate fatty acid levels to the efficacy of the therapy.

The following clinical outcome variables were assessed at baseline and after 6 months by the same calibrated examiner, using a University of North Carolina probe (UNC15, Hu-Friedy, Frankfurt, Germany):

- Full-mouth plaque score (FMPS)
- Full-mouth bleeding score (FMBS)
- Probing pocket depth (PPD)
- Gingival recession (REC)
- Clinical attachment level (CAL).

PPD, REC and CAL were measured for each site and then averaged. The obtained values were used for the following analyses.

The primary endpoint was the comparison of periodontal values between the two groups; the secondary endpoint was to evaluate if lipid levels had a direct effect on periodontal healing processes.

Assuming a two-sided type I error of 5 percent, we estimated that we would need 60 subjects (30 per group) for the study to have 80 percent power to demonstrate a statistically significant difference of 0.6 mm PPD variation between the control group and the experimental group.

Qualitative variables were described as absolute and percentage frequencies, while quantitative variables were summarized as mean, median, minimum, maximum and standard deviation.

TGR, LDL, HDL and TCH were treated as categoric variables with pre-defined classes (TG1 = Triglyceride levels under 150 mg/dL; TG2 = Triglyceride levels between 150 mg/dL and 199 mg/dL; TG3 = Triglyceride levels between 200 mg/dL and 499 mg/dL; TG4 = Triglyceride levels over 500 mg/dL. LDL1 = Low-density lipoprotein under 100 mg/dL; LDL2 = Low-density lipoprotein over 100 mg/dL. HDL1 = High-density lipoprotein under 200 mg/dL; HDL2 = High-density lipoprotein over 200 mg/dL. TCH1 = Total cholesterol under 200 mg/dL; TCH2 = Total cholesterol over 200 mg/dL).

The within-group differences between T0 and T1 were assessed by a paired *t*-test for each outcome (FMPS, FMBS, CAL, PPD, REC). The comparisons between groups were conducted with an independent *t*-test for each outcome (FMPS, FMBS, CAL, PPD, REC). A multivariate linear regression model with a stepwise selection procedure was conducted for each outcome measure between T1 and T0 to account for the impact of all other recorded covariates (blood pressure, triglycerides, total cholesterol, HDL, LDL) and the differences between the two groups at baseline on the effects of an improved nutritional plan on periodontal healing.

All analyses were performed with the RStudio software (RStudio Team, Boston, MA, USA), and the threshold for statistical significance was set at 0.05.

## 3. Results

The sample consisted of 60 subjects; 32 (53%) were treated in the ND group and 28 (47%) in the OD group. A summary of demographic characteristics of the sample is presented in Table 1, and the results of the blood tests are presented in Table 2.

In both groups, the periodontal treatment improved the recorded periodontal outcomes (FMPS, FMBS, CAL, PPD); the values measured at baseline and at T1 are reported in Table 3. An increase of the REC, as a consequence of the periodontal treatment, was recorded. The intra-class comparisons, as conducted with a paired *t*-test, found that the differences between T0 and T1 were statistically significant in both groups. Although the outcomes showed a higher improvement in the OD Group, these inter-class differences were not statistically significant.

The linear regression model results are reported in Table 4. The optimized diet was associated with a higher reduction in PPD and FMBS after the treatment, while patients who had higher LDL levels (over 100 mg/mL) had a less favorable improvement of PPD. None of the recorded variables were significant for CAL, REC and FMPS changes from baseline.

The other recorded variables had no statistically significant impact on the recorded periodontal outcomes.

## 4. Discussion

Diet and nutrition are generally categorized as modifiable lifestyle risk factors for the development of periodontal disease [17].

Altering the intake of both macro- and micronutrients could, therefore, improve the sulcus inflammatory status. The pathogenesis of periodontitis states that the disease develops following the emergence of dysbiosis in susceptible individuals and that connective tissue damage and alveolar bone resorption are host-mediated as a direct consequence of the hyper-inflammatory process that originates in the periodontal pocket [13,18]. As such, many probiotics have been proposed as an adjunct to conventionally adopted chemical agents, such as chlorhexidine, as they could promote maintenance of eubiosis in the long term and reduce the pathological bacterial load [19,20,21].

Excess glucose derived from overnutrition leads to the formation of free fatty acids and triglycerides within adipocytes. Insulin production in response to excess carbohydrate levels also decreases the breakdown of fat (lipolysis) within adipose tissue, increasing adiposity. The adipose tissue is described in the literature as an endocrine organ capable of secreting adipocytokines, such as tumor necrosis factor (TNF), interleukin (IL)-6, IL-1, adiponectin and leptin. These adipocytokines also trigger ROS production in inflammatory cells, further enhancing inflammatory cascades and oxidative stress.

Chronic hyperglycemia is also associated with the production of advanced glycation end products (AGEs). Oxidative stress reduces the pancreatic beta-cell function and intracellular antioxidant capacity and can lead to the development of insulin resistance. Thus, chronic hyperglycemia-induced oxidative stress over time causes insulin resistance, further elevating plasma glucose levels.

Several studies in the literature identified the role of oxidative stress in the pathobiology of chronic periodontitis. Increased levels of biomarkers for tissue damage induced by ROS have been observed in periodontitis patients and in response to oxidative stress, antioxidant enzymes appear upregulated in inflamed periodontal tissues and gingival crevicular fluid and seem to be correlated inversely with pocket depth.

In this study, the efficacy of applying an improved diet plan as an adjunctive therapy on treating periodontitis was assessed. Periodontal outcomes improved in both groups as an effect of the periodontal therapy. The application of an improved diet significantly influenced FMBS and PPD, which are directly related to the inflammatory levels present in the periodontal pocket.

Given the diet plan’s effects on reducing PPD and FMBS, which are significant prognostic factors, a reduction of the progression of the disease should be expected. However, the prevalence of periodontitis seems to have remained virtually constant during the past 3000 years in Great Britain, despite considerable changes in the oral environment. In an isolated community (Isla Grande, Colombia) with no dental services and a low education level, a community periodontal index of treatment needs (CPITN) score of one (presence of bleeding on probing (BOP)) was found in only 18% of subjects and 11% presented with probing depths (PDs) ‡5 mm (CPITN = 4) [6].

However, high glucose and lipid levels may generate ROS, overcoming endogenous antioxidant systems and as a result, causing oxidative stress. Multiple elevations in glucose levels can lead to the development of chronic inflammatory pathologies, such as coronary artery disease.

Longitudinal studies are needed to confirm whether successful periodontal therapy associated with other adjunctive therapies may influence the oxidative and nonoxidative inflammatory burden within the peripheral vasculature.

During the diagnostic phase, the clinician should try to consider the oral cavity and identify all factors that can potentially help the patient reach the treatment goal of prevention or management of periodontal disease and, thereby, possibly improve general health [2].

Studies with longer follow-up times are needed to confirm these observations and to relate the effect of non-surgical periodontal therapy associated with an improved diet plan on inflammation markers and antioxidant enzymes as well as on other clinical markers (e.g., tooth loss) that better define the socioeconomic effects of periodontitis.

Our report presents some flaws: the included patients were not randomly assigned to the two allocation groups (a patient’s choice of following an optimized diet may be related to their compliance, which plays a key role in the treatment of periodontal disease) and the clinicians were not blinded to which group patients were allocated. In addition, we did not monitor the glycemic status of the included patients, which could have a major effect on our recorded data and therefore alter our results, given the well-known relationship between glucose levels and periodontitis. To our knowledge, this is the first study to have observed the effects of the application of a diet plan on the efficacy of periodontal treatment, outlining that a diet rich in macro- and micronutrients with anti-inflammatory capabilities can reduce the clinical signs of periodontal disease.

## 5. Conclusions

In the present study comparing the clinical efficacy of performing non-surgical periodontal therapy alone or associated with an optimized diet as adjunctive therapy, the application of an improved diet plan achieved an increased reduction in PPD and FMBS after non-surgical periodontal therapy than after periodontal treatment alone. More studies are needed to observe the long-term effects on other markers of periodontal disease.

## Figures and Tables

**Table 1:** The demographic characteristics of the sample.

	ND ^b^	OD ^c^
Age (years)	51.59 ± 12.90	50.86 ± 11.69
Gender	15 Males, 17 Females	12 Males, 16 Females
Weight (kg)	78.19 ± 11.63	79.82 ± 11.82
Height (cm)	172.1 ± 9.30	172.3 ± 8.62
Bmi ^a^	26.34 2.83	26.78 ± 2.44
Ethnicity		
Afro-American	2	1
Asian	1	3
Caucasian	27	23
Latino-American	2	1
Number of Teeth	25.06 ± 2.67	25.46 ± 2.89

^a^ = Body Mass Index; ^b^ = Non-optimized diet; ^c^ = Optimized diet.

**Table 2:** Clinical characteristics of the sample at baseline.

		ND ^k^	OD ^l^
Blood pressure	1	9	10
	2	23	18
Triglycerides	TG1 ^a^	13	14
	TG2 ^b^	12	7
	TG3 ^c^	6	7
	TG4 ^d^	1	0
Total cholesterol	TCH1 ^e^	16	13
	TCH2 ^f^	16	15
HDL	HDL1 ^g^	18	13
	HDL2 ^h^	14	15
LDL	LDL1 ^i^	14	13
	LDL ^j^	18	15

^a^ = Triglycerides levels under 150 mg/dL; ^b^ = Triglycerides levels between 150 mg/dL and 199 mg/dL; ^c^ = Triglycerides levels between 200 mg/dL and 499 mg/dL; ^d^ = Triglycerides levels over 500 mg/dL; ^e^ = Total cholesterol under 200 mg/dL; ^f^ = Total cholesterol over 200 mg/dL; ^g^ = Low-Density Lipoprotein under 100 mg/dL; ^h^ = Low-Density Lipoprotein over 100 mg/dL.; ^i^ = High-Density Lipoprotein under 200 mg/dL; ^j^ = High-Density Lipoprotein over 200 mg/dL; ^k^ = Non-optimized diet; ^l^ = Optimized diet.

**Table 3:** The periodontal markers, as measured at baseline after six months and the difference between the two values.

	ND ^D^			OD ^E^	
	T0	T1	T1-T0	T0	T1
CAL ^a^	4.47 ± 0.92	3.78 ± 0.94	−0.69 ± 0.59 **	4.96 ± 0.96	4.07 ± 0.94
PPD ^b^	3.97 ± 0.82	3.03 ± 0.82	−0.94 ± 0.67 **	4.25 ± 0.89	3.00 ± 0.54
REC ^c^	0.47 ± 0.51	0.75 ± 0.72	0.28 ± 0.58 *	0.68 ± 0.82	1.07 ± 0.90
FMPS ^d^	65.28 ± 12.86	20.94 ± 4.29	−44.34 ± 14.03 **	66.61 ± 11.47	21.57 ± 4.62
FMBS ^e^	70.88 ± 10.43	24.5 ± 2.86	−46.38 ± 11.65 **	67.93 ± 9.47	22.64 ± 3.09

^a^ = Clinical Attachment Level; ^b^ = Periodontal Probing Depth; ^c^ = Gingival Recession; ^d^ = Full-Mouth Plaque Score; ^e^ = Full-Mouth Bleeding Score; ^D^ = Non-optimized diet; ^E^ = Optimized diet * = Intra-class comparison by paired *t*-test (*p*-value < 0.05); ** = Intra-class comparison by paired *t*-test (*p*-value < 0.001).

**Table 4:** The results of the regression analyses.

Dependent Variable	Independent Variable	Coefficient
FMBSD ^a^	Group Optimized diet	−1.88
PPDD ^b^	Group Optimized diet	−0.34
	LDL levels ^c^ Under 100 mg/dL	−0.82

^a^ = Full-Mouth Bleeding Score difference between T1 and T0; ^b^ = Pocket probing depth difference between T1 and T0; ^c^ = Low-Density Lipoprotein.

## Data Availability

The data presented in this study are available on request from the corresponding author.
